# Heterozygous Ldlr-Deficient Hamster as a Model to Evaluate the Efficacy of PCSK9 Antibody in Hyperlipidemia and Atherosclerosis

**DOI:** 10.3390/ijms20235936

**Published:** 2019-11-26

**Authors:** Yue Wu, Ming-Jiang Xu, Zhiyou Cao, Chun Yang, Jinjie Wang, Bijue Wang, Jian Liu, Yuhui Wang, Xunde Xian, Fang Zhang, George Liu, Xiaoli Chen

**Affiliations:** 1 Novo Nordisk Research Centre China, Novo Nordisk A/S, Beijing 102206, China; utopianwy@hotmail.com (Y.W.); mingjiangxu@gmail.com (M.-J.X.); zhca@novonordisk.com (Z.C.); bw@novonordisk.com (B.W.); willian85@163.com (J.L.); fazn@novonordisk.com (F.Z.); 2 Institute of Cardiovascular Sciences, Key Laboratory of Molecular Cardiovascular Sciences, Ministry of Education, Peking University, Beijing 100191, China; bjmuyc@bjmu.edu.cn (C.Y.); wangjinjie1994@126.com (J.W.); wangyuhui2009@bjmu.edu.cn (Y.W.); xianxunde@163.com (X.X.)

**Keywords:** dyslipidemia, animal model, low-density lipoprotein, low-density lipoprotein receptor, proprotein convertase subtilisin/kexin type 9, atherosclerosis

## Abstract

Proprotein convertase subtilisin/kexin type 9 (PCSK9) plays a key role in cholesterol homeostasis and atherogenesis. However, there are only limited rodent models, with a functional low-density lipoprotein receptor (LDLR) pathway and cholesteryl ester transfer protein (CETP) to evaluate the drug candidates targeting the PCSK9/LDLR pathway, that are translatable to humans. Here, by using our recently generated LDLR heterozygote (*Ldlr+/−*) hamster model with functional LDLR pathway and CETP function, we seek to evaluate the effect of a PCSK9 antibody, evolocumab, on dyslipidemia and atherosclerosis compared with ezetimibe, an effective inhibitor of cholesterol absorption, as a positive therapeutic control. We show that the plasma levels of total cholesterol (TC), low-density lipoprotein cholesterol (LDL-C), and triglyceride (TG) were significantly increased in *Ldlr+/−* hamsters fed a high-fat high-cholesterol (HFHC) diet; therefore, areas of atherosclerotic lesion in the aorta were obviously increased and positively correlated with plasma LDL-C and TC. Circulating free PCSK9 was downregulated by the HFHC diet and was undetectable in the evolocumab treated group, as expected. Most importantly, either evolocumab or ezetimibe treatment prevented HFHC diet-induced hyperlipidemia and subsequent atherosclerotic plaque formation. The results indicate that *Ldlr*+/− hamsters fed an HFHC diet represent an ideal rodent model to evaluate drug candidates that affect LDLR pathways.

## 1. Introduction

Familial hypercholesterolemia (FH) is a genetic disorder caused by a mutation in the low-density lipoprotein receptor (LDLR) gene, which makes the body unable to uptake LDL cholesterol (LDL-C) from the blood. This leads to severe elevation in LDL-C levels and results in advanced atherosclerotic plaque formation, coronary artery disease (CAD), and death [1].

Currently, *Ldlr* knockout (KO) mouse and apolipoprotein E (*Apoe*) KO mouse models are the two widely used hypercholesterolemia and atherosclerosis rodent models [2]. However, both animal models poorly characterize the rapid and lethal progression of CAD in FH patients. Moreover, these two animal models are deficient in the key lipid metabolism pathway, the LDLR pathway, which largely limits their application in drug target discovery and validation targeting the LDLR pathway. Furthermore, mice do not express cholesteryl ester transfer protein (CETP), which is the key enzyme in lipid and cholesterol transport. These shortages in mouse models are partially offset in APOE*3Leiden.*CETP* mice, which have human CETP and human mutant APOE3*Leiden [3]. In APOE*3Leiden.CETP mice fed a Western diet, PCSK9 antibody treatment significantly lowers total cholesterol (TC) and triglyceride (TG) levels, as well as atherosclerotic lesion areas, which are not observed in either *Apoe* KO or *Ldlr* KO mice [4]. However, the lipoprotein profiles of APOE*3Leiden.CETP mice are significantly different from those of humans due to hepatic ApoB editing in murine species; therefore, they express ApoB48 in very-low-density lipoprotein (VLDL) [5]. Since increased apoB100-containing lipoproteins is a dominant risk factor of atherosclerosis and CAD in FH patients, it is important that animal models closely mimic the human lipoprotein profile.

More recently, *Ldlr*-deficient Syrian hamster models have been generated [6]. In combination with a high-fat high-cholesterol (HFHC) diet, the heterozygous *Ldlr (Ldlr+/−)* hamsters showed marked hypercholesterolemia and hypertriglyceridemia as early as 1 week after feeding, and time-dependently increased atherosclerotic lesions in aorta from 2 to 4 months [6,7]. Different from mice, hamsters express CETP, and as such, among rodents, they have similar lipid metabolism pathways to humans. The plasma lipid profiles of *Ldlr+/−* and *Ldlr−/−* hamsters are very similar to those of heterozygous FH (HeFH) and homozygous FH (HoFH) patients with *LDLR* mutations [8], which makes hamsters a useful rodent model for evaluating atherosclerosis. Most importantly, *Ldlr+/−* hamsters have LDLR expression in the liver; therefore, the model has the potential to be used for evaluating agents targeting the LDLR pathway.

Proprotein convertase subtilisin/kexin type 9 (PCSK9) is a hepatic protease that internalizes LDLR into lysosomes and results in the degradation of LDLR [9]. It has also been demonstrated that PCSK9 can induce a pro-inflammatory response in macrophages [10]. Individuals carrying a nonsense mutation of PCSK9 have reduced plasma LDL-C levels and lower incidences of coronary events [11,12]. As such, PCSK9 inhibition has become a very promising strategy for the prevention of cardiovascular events. Since 2015, PCSK9 antibodies have been approved for treating primary hyperlipidemia by reducing plasma LDL-C, and more PCSK9 inhibitors are under development. The FOUTIER trial demonstrated that evolocumab reduced myocardial infarction, stroke, and coronary revascularization by more than 20% [13]. Furthemore, PCSK9 antibodies also improve the arterial steffness [14]. Although there are emerging new drug candidates targeting PCSK9, there has been no atherosclerosis animal model that is translatable to humans to evaluate the role of the PCSK9-LDLR pathway in atherosclerosis.

The current study is conducted to evaluate the preventive effect of a PCSK9 antibody, evolocumab, on plaque formation in atherosclerotic-prone *Ldlr+/−* hamsters fed an HFHC diet. Since the source of cholesterol is mainly from diet in this model, ezetimibe, which acts by decreasing cholesterol absorption in the small intestine, was used as a positive therapeutic control.

## 2. Results

### 2.1. Ldlr+/− Hamsters Displayed Dyslipidemia Compared with WT Hamsters when Fed with Chow Diet

**Figure 1:**
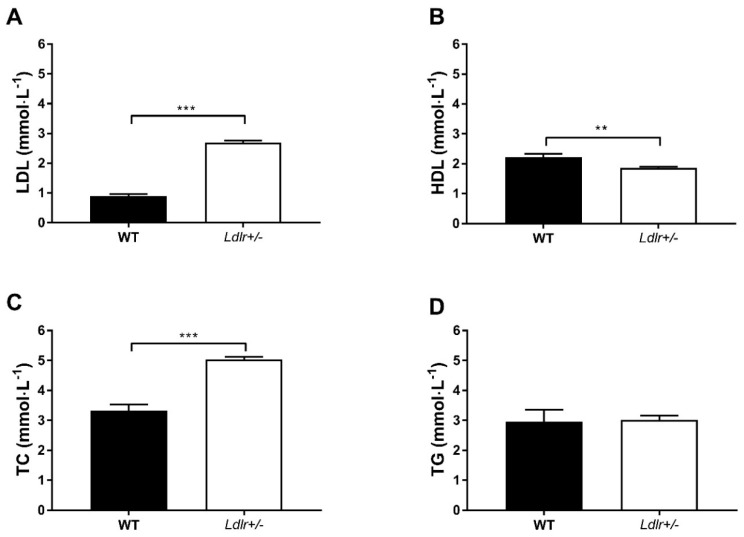
Lipid profiles of wild-type (WT; N = 9) and *Ldlr+/−* (N = 44) hamsters fed with chow diet. ** The concentrations of plasma LDL (**A**), HDL (**B**), TC (**C**) and TG (**D**) were measured in WT and *Ldlr*+/- hamsters. *p* < 0.01, *** *p* < 0.001. An unpaired *t*-test was performed. LDL, low-density lipoprotein; HDL, high-density lipoprotein; TC, total cholesterol; TG, triglyceride.

Before feeding the *Ldlr+/−* hamsters with the HFHC diet, their baseline blood lipid levels were pooled together (N = 44) and compared with the levels of WT hamsters (N = 9; Figure 1). At the age of 2 to 3 months, the LDL-C and TC levels of *Ldlr+/−* hamsters were 3 times (*p* < 0.001) and 1.5 times (*p* < 0.001) the levels of WT hamsters, respectively. On the other hand, the *Ldlr+/−* hamsters had slightly lower HDL-C levels compared to WT hamsters (*p* < 0.01). There was no difference in TG levels between *Ldlr+/−* and WT hamsters.

### 2.2. Evolocumab or Ezetimibe Treatment Reduced HFHC Diet-Induced Hyperlipidemia

**Figure 2:**
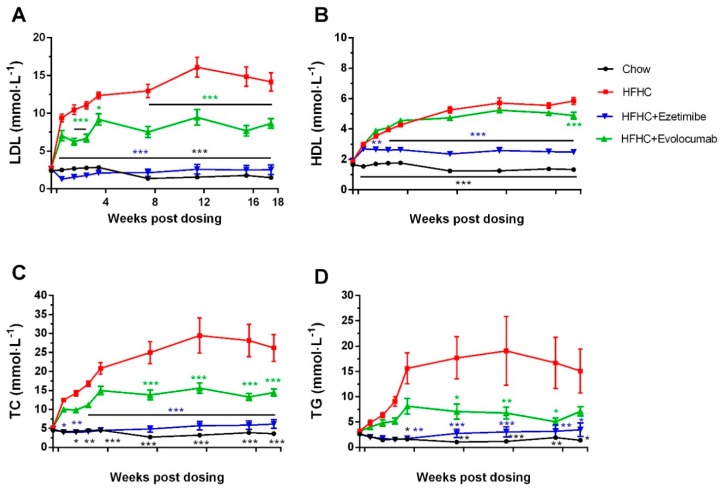
Evolocumab or ezetimibe treatment improved high-fat high-cholesterol (HFHC) diet-induced dyslipidemia in *Ldlr+/−* hamsters. *Ldlr+/* hamsters were fed with chow or HFHC diet for 18 weeks, with simultaneous administration of ezetimibe or evolocumab. Blood samples were obtained at indicated time points. Plasma (**A**) LDL-C, (**B**) HDL-C, (**C**) TC, and (**D**) TG were measured. * *p* < 0.05, ** *p* < 0.01, *** *p* < 0.001 compared with values in HFHC model group. Two-way ANOVA with Dunnett’s multiple comparisons test was performed.

HFHC diet feeding increased plasma TC, TG, LDL-C, and HDL-C levels from day 3 in *Ldlr+/−* hamsters, the levels gradually increased in the following 12 weeks and then had a decreasing trend (Figure 2). In week 12, LDL-C and TC levels increased ~500% in the HFHC diet group. Evolocumab prevented HFHC diet-induced LDL-C, TC, and TG increase by 40%–60% (Figure 2A,C,D), but had no or less effect on HDL-C (Figure 2B). Ezetimibe almost completely abolished the increases of LDL-C, TC, and TG caused by the HFHC diet. The HDL-C level in the ezetimibe treated group was 43% of that of the HFHC diet model group and 187% of that of the chow diet control group (Figure 2B).

### 2.3. Evolocumab or Ezetimibe Treatment Prevented HFHC Diet-Induced Atherosclerotic Plaque Formation in Aorta

**Figure 3:**
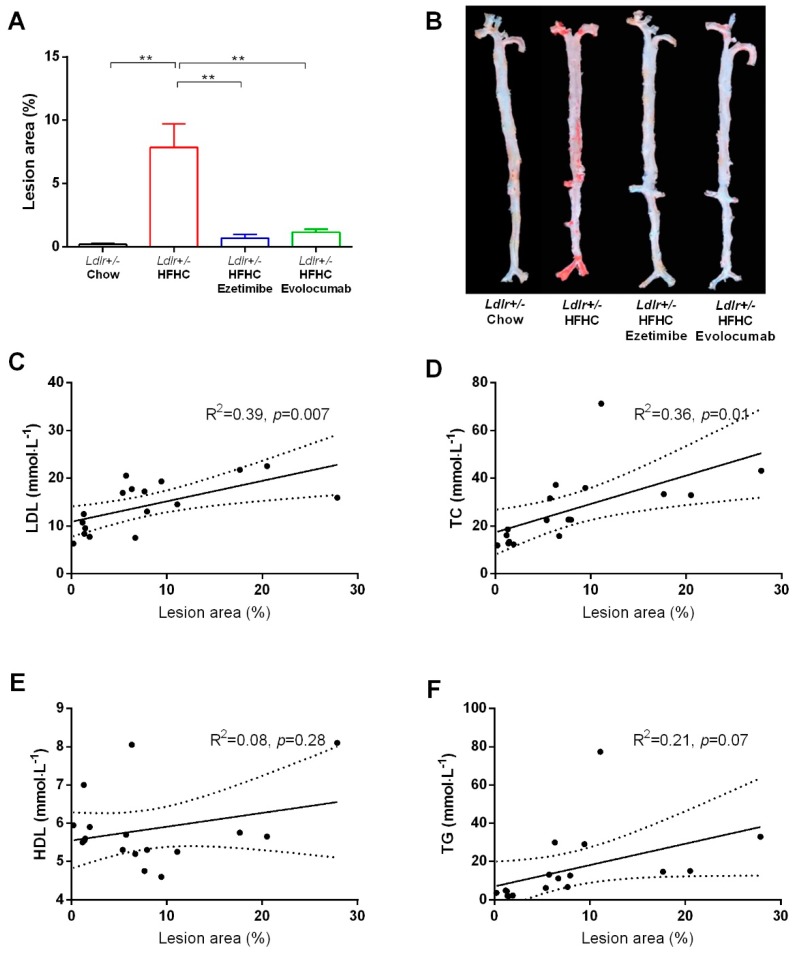
En face analysis of aortic lesion area and its correlation with plasma lipid profiles. (**A**) Quantitative analysis of whole aorta lesion area. ** *p* < 0.01 compared with values in the HFHC diet model group. One-way ANOVA with Dunnett’s multiple comparisons test was performed. (**B**) Representative images of en face analysis. (**C**–**F**) Correlations between lesion areas and plasma LDL-C, TC, HDL, and TG in the HFHC diet model group were analyzed by linear regression. Dashed line shows 95% confidence band of best fit line.

The *Ldlr+/−* hamsters fed with chow diet for 18 weeks did not develop aortic plaque, while those fed with the HFHC diet developed obvious atherosclerotic plaque (ranging from 0.2% to 27.8%, mean value 7.9%) in the aortic arch, abdominal aorta, and iliac aorta, evidenced by en face staining (Figure 3A,B). HFHC diet-induced atherosclerotic plaque formation in evolocumab and ezetimibe treated groups was significantly decreased (ranging from 0.3% to 2.4%, mean value 1.2%; and from 0.1% to 3.1%, mean value 0.7%, respectively) (Figure 3A,B). Further correlation analysis of the HFHC diet control group showed that the lesion area was positively correlated with plasma LDL-C and TC levels at termination (Figure 3C,D), but not with plasma HDL-C and TG levels (Figure 3E,F).

### 2.4. Evolocumab or Ezetimibe Treatment Prevented HFHC Diet-Induced Atherosclerotic Lesions in Aortic Root

**Figure 4:**
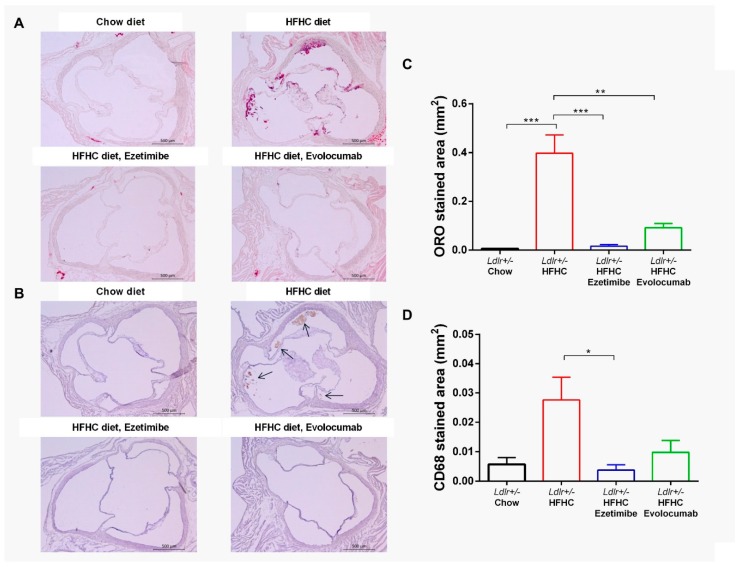
Evolocumab or ezetimibe treatment prevented HFHC diet-induced atherosclerotic lesions in aortic valves. (**A**) Representative images of Oil Red O (ORO) staining. (**B**) Representative images of CD68 immunohistochemical staining. Arrowheads indicate CD68 immunohistochemical stained areas. (**C**) Quantified lesion area of Oil Red O staining. (**D**) Quantified CD68-positive staining area. * *p* < 0.05, ** *p* < 0.01, *** *p* < 0.001, compared with values in the HFHC diet model group. One-way ANOVA with Dunnett’s multiple comparisons test was performed.

Aortic root cross-sectioning and immunohistochemical staining were done to investigate the atherosclerotic lesions in aortic valves and the components in the plaque. Similar to the en face analysis, the *Ldlr+/−* hamsters fed the HFHC diet had significantly increased lesion areas in aortic valves compared to chow diet controls measured by image analysis of Oil Red O staining, and the atherosclerotic development was prevented by either evolocumab or ezetimibe treatment (Figure 4A,C). The plaque in the HFHC diet group was CD68-positive (a macrophage marker) and was also significantly decreased by evolocumab or ezetimibe treatment (Figure 4B,D). There were no differences in the four groups with regard to Masson’s trichrome and SMA-α staining (Supplementary Figure S2).

### 2.5. Plasma-Free PCSK9 Level was Decreased by HFHC Diet and Completely Removed by Evolocumab Treatment

**Figure 5:**
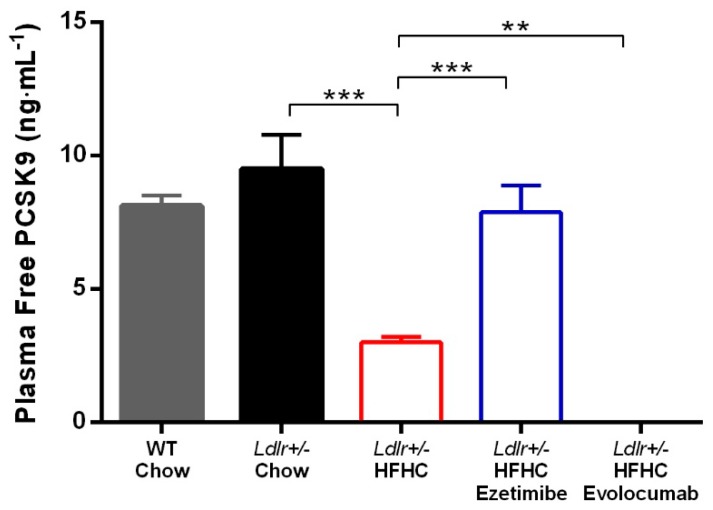
Plasma-free PCSK9 protein concentration. ** *p* < 0.01, *** *p* < 0.001 compared with values in the HFHC model group. One-way ANOVA with Dunnett’s multiple comparisons test was performed.

When binding with PCSK9 antibodies, plasma PCSK9 protein will not bind to LDLR and facilitate its degradation. As such, we developed a LOCI assay to detect free functional plasma PCSK9 levels. When *Ldlr+/−* hamsters were fed a chow diet, their plasma-free PCSK9 levels did not differ from those of WT hamsters. However, HFHC diet feeding significantly reduced plasma-free PCSK9 levels in *Ldlr+/−* hamsters. Ezetimibe treatment prevented the reduction of plasma-free PCSK9 levels. With chronic evolocumab treatment, plasma-free PCSK9 protein was undetectable in this assay (LLOQ = 0.2 ng·mL^−1^) at termination (3 days after last dosing) (Figure 5).

## 3. Discussion

HeFH patients have increased plasma TC and LDL-C levels, as well as normal TG and HDL-C levels [1], which is similarly observed in the *Ldlr+/−* hamsters at baseline (Figure 1), but not in mouse atherosclerosis models, such as *Apoe* KO and *Ldlr* KO mouse models. This gene titration effect indicates that the plasma lipid profiles and lipid metabolism pathways of *Ldlr+/−* hamsters resemble those of HeFH patients. The atherosclerotic lesions of *Ldlr+/−* hamsters fed an HFHC diet were mainly found in the aortic arch and abdominal aorta (Figure 3), and more importantly also in the coronary artery [8], which is similar to human atherosclerosis. In contrast, atherosclerotic lesions that form in mouse models are mainly found in the aortic root and aortic arch, but not in the abdominal aorta and coronary artery [15,16]. These findings demonstrate that the *Ldlr+/−* hamster model better resembles the human atherosclerosis condition compared to mouse models. The result from the current model is likely be more translatable to humans.

We further characterize the components and severity of the lesions by histopathology analysis. Oil Red O staining results in whole aortas and aortic roots demonstrate that the HFHC diet model group had significantly increased lesion areas compared to the chow diet control group (Figure 3 and Figure 4). Immunostaining results show that there was macrophage infiltration in the lesion areas, as stained by CD68 (Figure 4). However, there were no smooth muscle cells or fibrosis observed in the lesion areas, as stained by SMA-α and Masson’s trichrome (Supplementary Figure S2). These data indicate that the atherosclerotic lesions of the hamsters in the current study were still in the early stage. Further correlation analysis shows that lesion areas were positively correlated with plasma TC and LDL-C levels, but not TG and HDL-C levels (Figure 3), suggesting that the atherogenesis in *Ldlr+/−* hamsters is driven by high cholesterol. As such, the animals may have developed more severe or late-stage plaque lesions with increased cholesterol levels with the diet. It has been demonstrated that 1% of cholesterol in a high-fat diet is well tolerated by *Ldlr+/−* hamsters [8].

Plasma-free PCSK9 levels were measured in this study to characterize the binding of evolocumab to PCSK9 in vivo at steady state, as well as the influence of diet or ezetimibe treatment on PCSK9 expression. First, we found that HFHC diet significantly reduced the plasma PCSK9 protein level (Figure 5). Both LDLR and PCSK9 are transcriptionally regulated by sterol regulatory element-binding protein-2 (SREBP-2) [17]. Excessive cholesterol absorption and increased LDL-C lead to reduced *Srebp2* gene expression, and consequently decreased *Ldlr* and *Pcsk9* gene expression. It has been observed in rats fed a high-cholesterol diet (2%) that the liver mRNA expression of *Srebp2*, *Ldlr*, and *Pcsk9* was reduced by 50%, 30%, and 65%, respectively. The liver PCSK9 protein was reduced by 70%; however, the liver LDLR protein was increased by 70%. The author proposed that the discrepancies between gene expression and protein level of LDLR could be explained by posttranscriptional regulation by PCSK9 [18]. The same finding was reported in monkeys; after feeding with an HFHC diet (0.15% cholesterol) for 1 month, the plasma PCSK9 level was reduced by 38%, and it recovered to the baseline level after the monkeys were switched back to a regular diet for 2 months [19].

Furthermore, ezetimibe treatment prevents the plasma PCSK9 reduction induced by the HFHC diet in *Ldlr+/−* hamsters (Figure 5). Our finding is in line with what has been reported in monkeys fed an HFHC diet [19]. However, in clinical studies, ezetimibe treatment did not result in increased circulating PCSK9 levels [20]. The different findings between HFHC diet animal models and humans can be explained by the difference in cholesterol sources. The sources of cholesterol are de novo synthesis and dietary intake. In the current model as well as in most atherosclerosis animal models, a high-cholesterol diet is used to accelerate disease progression, and the excessive cholesterol in the circulation further suppresses cholesterol biosynthesis [21]. Ezetimibe works by directly inhibiting cholesterol absorption in the jejunal brush border [22,23]. In animal models with high cholesterol diet-induced atherosclerosis, the additional cholesterol absorbed from the diet is prevented by ezetimibe treatment, and thus the diet does not induce any reduction of plasma PCSK9, while in humans, the contribution of dietary cholesterol to TC is less, and thus the effect of ezetimibe is also minimal. Ezetimibe worked extremely well in preventing increased plasma cholesterol and TG as well as plaque formation in the current study. Though ezetimibe does not appear to block absorption of TG [24], the TG level of the ezetimibe treated group was significantly lower than that of the untreated group (Figure 2), which was also observed in other animal studies as well as in clinical trials [25,26]. The underlying mechanism is likely a secondary effect of reduced liver cholesterol accumulation. Clinical studies have shown that HeFH patients respond well to PCSK9 monoclonal antibodies [27]. Evolocumab reduced more than 50% of LDL-C and more than 40% of TC on the top of statin treatment in HeFH patients. The HDL-C level was not reduced by evolocumab treatment, and there was even an increase in the high dose group. More importantly, these clinical findings of PCSK9 antibodies were well reproduced in the current hamster study, where evolocumab treatment completely removed plasma-free PCSK9 and partially prevented increased LDL-C and TC during the 18 weeks of HFHC diet feeding. The LDL-C and TC levels of the evolocumab treated group were 40%–50% lower than those of the untreated group (Figure 2). Increased HDL-C level was not prevented by evolocumab treatment, which indicates a potential advantage of PCSK9 antibody therapy in subjects consuming an HFHC diet. However, the plasma LDL-C level of the evolocumab-treated group was still more than four times higher than that of the chow diet control group (Figure 2). The results show that complete abolishment of the functional plasma PCSK9 protein could not normalize the plasma LDL-C level induced by the HFHC diet, suggesting that combining PCSK9 antibody with other LDL-C-lowering drugs via different mechanisms would be necessary to further lower the LDL-C level in this population in order to reach the ideal level. Alirocumab and evolocumab did not reduce TG levels in HeFH patients; however, in the *Ldl+/−* hamsters fed an HFHC diet, the TG level was significantly elevated by the diet and reduced by evolocumab treatment (Figure 2). This disparity may be due to the fact that HeFH patients recruited in the clinical trials did not have hypertriglyceridemia [27]. Thus this finding may suggest that PCSK9 antibodies might also be used as TG-lowering agents in hypertriglyceridemia; however, this effect needs to be further proved in clinical trials. More importantly, although the LDL-C, TC, and TG levels in the evolocumab treated group were still higher than those in the chow diet control group, PCSK9 antibody treatment successfully prevented plaque formation in aortas and aortic valves (Figure 3 and Figure 4).

## 4. Material and Methods

### 4.1. Reagents and Diets

The recombinant human PCSK9 monoclonal antibody (immunoglobulin G2 (IgG2)), evolocumab, was produced at Novo Nordisk Research Center China, and phosphate-buffered saline (PBS) was used as vehicle. By targeting its epitope, evolocumab blocks the interaction between PCSK9 and repeat A of the epidermal growth factor homology (EGF-A) domain of LDLR [28,29]. The HFHC diet was made based on research diet D12450H with 20.0 kcal% protein, 44.5 kcal% carbohydrate, 35.5 kcal% fat, and 0.5 g% cholesterol. The HFHC diet and HFHC diet with 0.018 g% ezetimibe were purchased from Research Diet Inc. (NJ, USA).

### 4.2. Hamsters

Wild-type (WT) Syrian hamsters were purchased from Vital River Laboratories (Beijing, China), and *Ldlr+/−* hamsters were generated by Dr. George Liu’s lab at Peking University using CRISPR/Cas9 gene-editing technology [8]. Male WT and Ldlr+/− hamsters at age 10–12 week old were used in current study. The WT animals were only used for comparison of baseline lipid profiles and free PCSK9 levels with the *Ldlr+/−* hamsters, and An unpaired in the in vivo study. The animals were housed in rooms at room temperature with a 12/12 h light/dark cycle, with environmental enrichment added to the cages. Standard chow diet and water were available ad libitum prior to the study initiation. The animals were 12 weeks old when the study started. The animal study protocol was approved by the Institutional Animal Care and Use Committee of Novo Nordisk Research Center China, as well as the Ethical Review Council of Novo Nordisk A/S.

### 4.3. Animal Study Design

The animals were acclimatized at the Peking University Health Science Center animal facility for 2 weeks before the study started. The *Ldlr+/−* hamsters were divided into 4 groups. Evolocumab was dosed subcutaneously at 30 mg·kg^−1^ once weekly. Ezetimibe was added to the diet, and the dosage was about 0.67 mg·kg^−1^·day^−1^. The HFHC diet feeding and drug treatment started at the same time on day 0 and lasted for 18 weeks. Blood samples were taken sublingually under N_2_O/O_2_/isoflurane anesthesia on days −3, 3, 10, 17, 24, 52, 80, 108, and 122 (study termination).

### 4.4. Plasma TC, TG, LDL-C, and HDL-C Measurements

Plasma TC, TG, LDL-C, and high-density lipoprotein cholesterol (HDL-C) levels were determined by a Cobas501 auto biochemical analyzer, with reagents (cat no. 03038866322, 04399803190, 03039773190, and 20767107322) purchased from Roche Diagnostics GmbH (D-68298, Mannheim, Germany), and procedures referred to the manufacturer’s instructions.

### 4.5. Plasma-Free PCSK9 Measurement

Plasma-free PCSK9 protein concentration was determined by an in-house developed luminescent oxygen channeling immunoassay (LOCI). In brief, LOCI is a homogeneous bead-based assay. LOCI reagents included 2 latex bead reagents and biotinylated evolocumab, which was one of the antibodies in the sandwich. One of the bead reagents was generic (donor beads), coated with streptavidin and containing a photosensitive dye. The second bead reagent (acceptor beads) was coated with the other anti-PCSK9 polyclonal antibody making up the sandwich. During the assay, the 3 reactants combine with free PCSK9 to form a bead–aggregate–immune complex. Illumination of the complex released singlet oxygen from the donor beads, which channeled into the acceptor beads and triggered chemiluminescence, was measured in an EnVision plate reader. The amount of light generated was proportional to the concentration of free PCSK9 (but not necessarily linear). The standard curve was fitted using a 5-parameter logistic equation [30] (Supplementary Figure S1). The lower limit of quantification (LLOQ) concentration of this assay was 0.2 ng·mL^−1^, which was calculated by fitting the LLOQ signal in the standard curve. The LLOQ signal was calculated using the following equation:

LLOQ signal = mean signal of negative controls + 5 × standard deviation (SD) of negative controls

Reagents were purchased from Sigma Aldrich or PerkinElmer.

### 4.6. Histopathology Analysis

The animals were euthanized under N_2_O/O_2_/isoflurane anesthesia followed by cardiac perfusion of 50 mL 0.9% saline through the left ventricle. Whole aortas and hearts were isolated and fixed in formalin for 4 hours before being transferred to 20% sucrose solution overnight. Ten serial 8 μm thick cryosections of hamster aortas, beginning at the aortic sinus, were collected every 104 μm. Sections were stained with Oil Red O, anti-CD68 antibody (BA3638, Boster), anti-smooth muscle actin-α (SMA-α) antibody (ab5694, Abcam), and Masson’s trichrome stain (ab150686, Abcam). Images were quantified using the Visiopharm Integrator System (version 4.5.3).

For en face analysis, aortas were longitudinally opened from the heart to the iliac artery, and atherosclerotic lesions were stained with Oil Red O. Images of en face aortic lesions were analyzed with the Visiopharm Integrator System (version 4.5.3), and data are presented as percentage of total section/aorta area.

### 4.7. Statistics

The animal number was determined based on pilot study data and statistical power analysis to reach 80% statistical power with less than a 0.05 type I error (2-sided). The data in the text and figures are shown as mean ± SEM. Statistical tests are specified in figure legends. No data were excluded from the analysis. GraphPad Prism 7.04 was used for statistical analysis. *P*-values of 0.05 or less were considered statistically significant.

## 5. Conclusions

In conclusion, evolucumab and ezetimibe prevent atherosclerotic plaque formation and increase LDL-C, TC, and TG levels induced by HFHC diet feeding in *Ldlr+/−* hamsters. The *Ldlr+/−* hamster atherosclerosis model is more translatable to humans compared to mouse models with regard to lipoprotein profiles, lipid metabolism pathways, and sites of lesions in the aorta. It can be used to evaluate therapeutic targets involving LDLR pathways.

## Supplementary Materials

Supplementary Materials can be found at https://www.mdpi.com/1422-0067/20/23/5936/s1.
