# Supplementary material for: Heterozygous Ldlr-Deficient Hamster as a Model to Evaluate the Efficacy of PCSK9 Antibody in Hyperlipidemia and Atherosclerosis
Source: Int J Mol Sci. 2019 Nov 26;20(23):5936. doi: 10.3390/ijms20235936 (PMC6929182; doi:10.3390/ijms20235936)
Supplement: Supplementary file 1 [file ijms-20-05936-s001.pdf]

Supplementary Materials

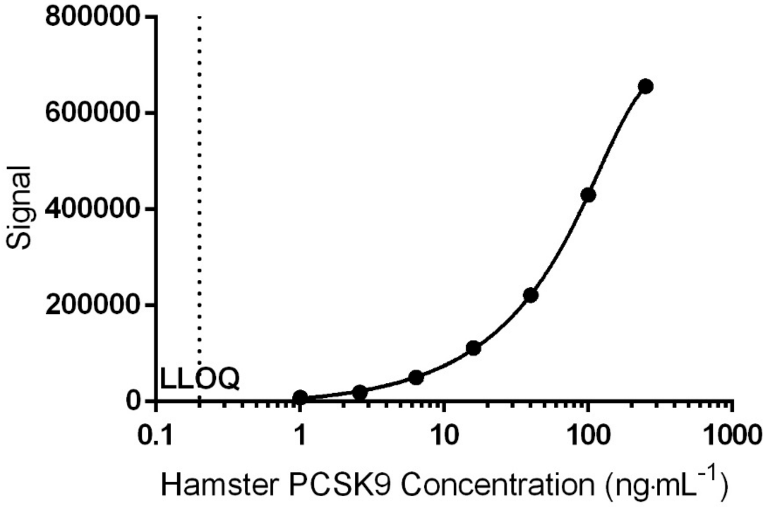

Supplementary Figure S1. Hamster free PCSK9 luminescent oxygen channeling immunoassay (LOCI) assay standard curve. Standard curve was fitted using five-parameter logistic equation. Lower limit of quantification (LLOQ) concentration of this assay was 0.2 ng·mL^{-1}.

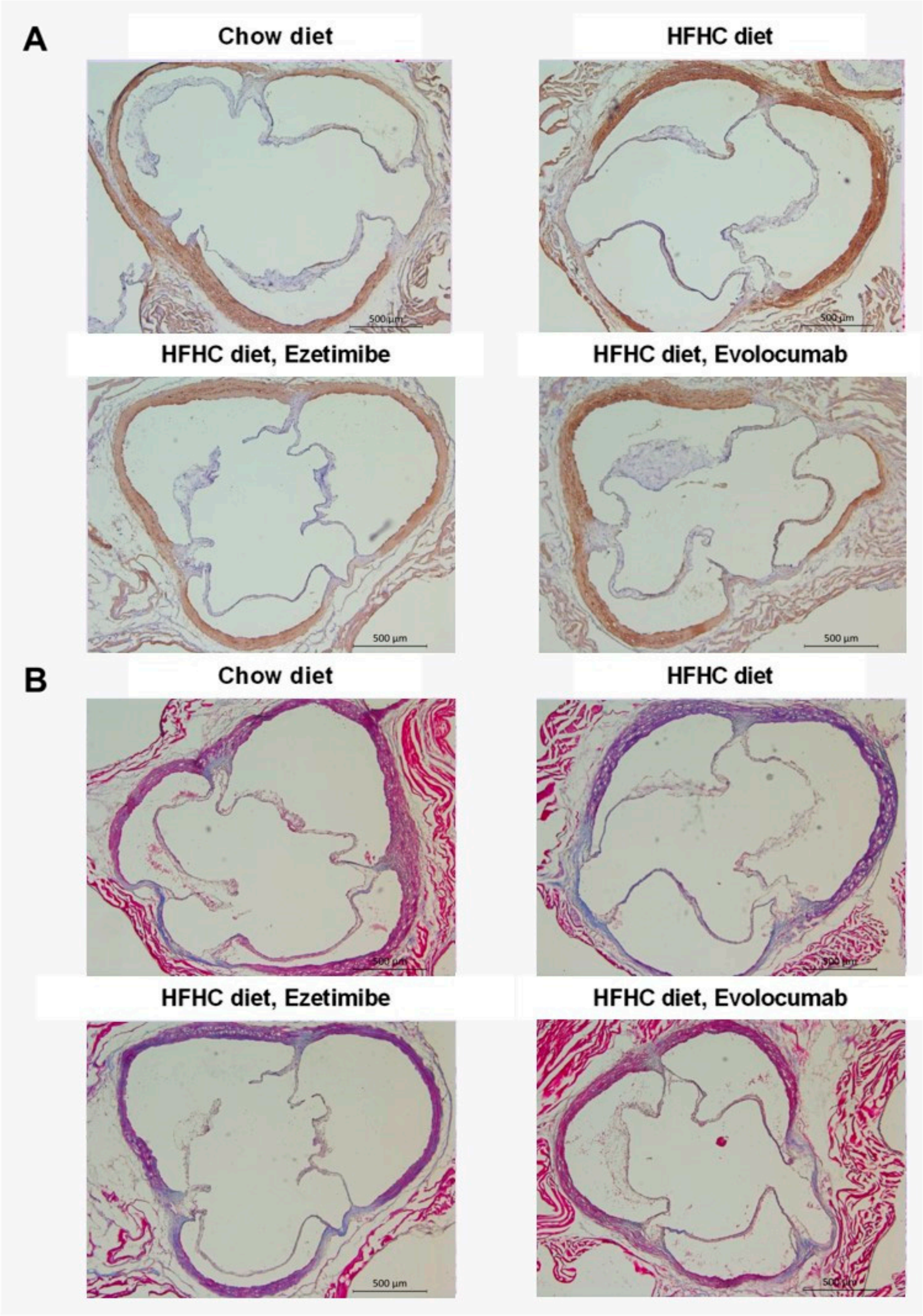

Supplementary Figure S2. Representative images of (**A**) smooth muscle actin-α (SMA-α) staining and (**B**) Masson's trichrome staining in aortic valve.
